# Recognition of emotional and expressive movements (gestures) and self-esteem of adolescents with affective disorders

**DOI:** 10.1192/j.eurpsy.2022.1078

**Published:** 2022-09-01

**Authors:** M. Zvereva, S. Avdyukhina

**Affiliations:** 1 FSBSI MENTAL HEALTH RESEARCH CENTER, Clinical Psychology, Moscow, Russian Federation; 2 Private practice, Private Practice, Moscow, Russian Federation

**Keywords:** affective disorder, self-esteem, gestures recognition, adolescent

## Abstract

**Introduction:**

Successful adolescence depends on ability to correctly understand emotionally expressive gestures, especially symbolic (same meaning for everyone) and expressive (individual understanding). Presence of an internal mismatch in adolescent’s self-esteem between what he shows in society and what he really feels can lead to difficulties in forming an adequate adult self-esteem.

**Objectives:**

Adolescents with affective disorders (F31) -12, normal adolescents - 32. Ages 12-17.

**Methods:**

Recognition of emotionally expressive movements: postures&gestures (gestures-test), direct self-esteem by Dembo-Rubinstein test and indirect self-esteem by color attitude test by Etkind.

**Results:**

The Mann-Whitney test showed significant differences between samples in terms of self-esteem gap - “mind” (U=270,000, p<0.37), “character” (U=279,000, p<0.20), “happiness” (U=288,000, p<0.01 ), gestures-test “symbolic” (U=301,000, p<0.003), “expressive” (U=292,000, p<0.007), “emotions” (U=109,000, p<0.028). Cluster analysis divided each of groups into two distinct clusters. Normal: Cluster1 small self-esteem gap, good gesture recognition, negative pole of emotions prevails. Cluster2 small self-esteem gap, worse gesture recognition, pole of emotions is closer to positive. Affective: Cluster1 large self-esteem gap in “mind”, good gesture recognition. Cluster2 large self-esteem gap in “character”, good gesture recognition and bright negative pole of emotions.

**Conclusions:**

Gestures recognition in normal group is significantly higher than in affective disorder group. Normal adolescents clusters are distinguished by change in gaps throughout self-esteem and pole of emotional recognition. Affective disorder clusters differ by significant gap in one of self-esteem parameters, as well as in the degree of emotional recognition. Those with the largest “character” gap are more likely to attribute negative emotions to gestures than those with larger “mind” gap.

**Disclosure:**

No significant relationships.

